# The Change of Self-Rated Health According to Working Hours for Two Years by Gender

**DOI:** 10.3390/ijerph15091984

**Published:** 2018-09-11

**Authors:** Jia Ryu, Yeogyeong Yoon, Hyunjoo Kim, Chung won Kang, Kyunghee Jung-Choi

**Affiliations:** 1 Department of Occupational and Environmental Medicine, College of Medicine, Ewha Womans University, Seoul 07985, Korea; jiyajiya000@gmail.com (J.R.); ygyoon319@naver.com (Y.Y.); 2 Department of Occupational and Environmental Medicine, Ewha Womans University Mokdong Hospital, Seoul 07985, Korea; hyunjoo@ewha.ac.kr (H.K.); scmfer@hanmail.net (C.w.K.)

**Keywords:** working hours, self-rated health, gender

## Abstract

Objective: The aim of this study was to confirm the association between working hours and self-rated health, and to find the degree of changes in health level by working hours according to gender. Methods: This study was based on the 929 workers (571 men and 358 women) from the Korean Labor and Income Panel Study during 2004–2006. To minimize the healthy worker effects, the study subjects included only those who did not have any chronic diseases, and who answered their health status as “moderate” or above in the baseline. Logistic regression analysis was used to confirm the associations between working hours and self-rated health. Results: In men, working hours per week of 47–52 h, 53–68 h, and >68 h were associated with 1.2, 1.3, and 1.1 times increases, respectively, in the odds ratio on worsened self-rated health, compared with the reference group (40–46 h). On the other hand, the risks were 1.0, 2.2, and 2.6 times increases in women. However, the results were different according to gender in the group with less than 40 h. The men with less than 40 h had a 0.9 times odds ratio on worsened self-rated health. For the women with less than 40 h, the odds ratio on self-rated health was 5.4 times higher than the reference group. Conclusions: Working more than 52 h per week had a negative effect on health, regardless of gender. However, in the group with less than 40 h, the negative association between working hours and self-rated health were shown only in women. Health outcomes due to working hours may differ by gender. Therefore, further studies are needed to explore the causes of these results.

## 1. Introduction

The working hours in South Korea are almost the longest in the world. According to the Organization for Economic Cooperation and Development (OECD) report, as of 2016, South Korea’s annual number of working hours (2069 h) was the third longest, preceded by Mexico (2255 h) and Costa Rica (2212 h). It is 306 h more than the average of OECD member countries, and 356 h longer than Japan [1].

Previous studies reported on the negative effects of working hours on health, but there are inconsistencies in the results. In South Korea, long working hours are associated with cardiovascular disease [2,3,4], suicidal ideation [5], depressive symptoms [6,7], and self-rated health [8,9]. Other countries confirmed that working hours correlated with deterioration of health status, including cardiovascular disease [10,11,12], diabetes [13], health behavior [14], depressive symptoms [15], and self-rated health [16,17].

Most studies show that self-rated health is an indicator that is closely related to mortality. That is, self-rated health serves as an independent predictor of mortality after adjusting for certain health status indicators [18]. However, until recently, the research on the working hours and self-rated health has been insufficient, and there have been limitations in obtaining a consistent conclusion [8,9]. The study found that the self-rated health (SRH) was worsened only in women with long working hours [8]. 

There may be several reasons for this discrepancy. Firstly, the reference points for categorizing working hours vary in each study. One study sets the reference point on the group who worked from 35 to 45 h per week [10]. Other studies have set the reference points at 52 h [17] and less than 40 h [19], to identify the associations between working hours and health. Secondly, it is possible to have a reverse-causality between working hours and health. There is insufficient consideration of the possible healthy-worker effects that are often encountered in studies. Some of the related studies also point to this as a major limitation [20,21]. Thirdly, it lacks consideration of effect modifiers, such as gender and occupation. Gender and occupation can act as a determinant of working hours. It is also a factor that may change the health effects of working hours. However, few studies have considered these factors together. Furthermore, in South Korea, the labor market is highly gender-segmented, and there is a great difference in the quality of work that men and women perform [22].

Therefore, we tried to confirm further evidence for the consistency of research on the association between working hours and self-rated health, after overcoming the aforementioned limitations. 

## 2. Materials and Methods

### 2.1. Study Subjects

We used data from the Korean Labor and Income Panel Study (KLIPS), which is an ongoing longitudinal study in South Korea. The KLIPS was designed to examine the various social and economic status, including employment status, income, education level, and occupation, for the same individuals annually. Among these, the data from the KLIPS between the 4th year (2001) and the 6th year (2003) were used in this study. The 4th year data was the only one that had a detailed survey of the respondents’ health status. The questionnaire on self-rated health was conducted in the 6th year survey, but not in the 5th year survey. For this reason, there was a limitation that the data was relatively old, but the data of those years was used. The KLIPS sample retention rate was 77.3% in the 4th year (2001), and also in the 6th year (2003) [23].

**Figure 1:**
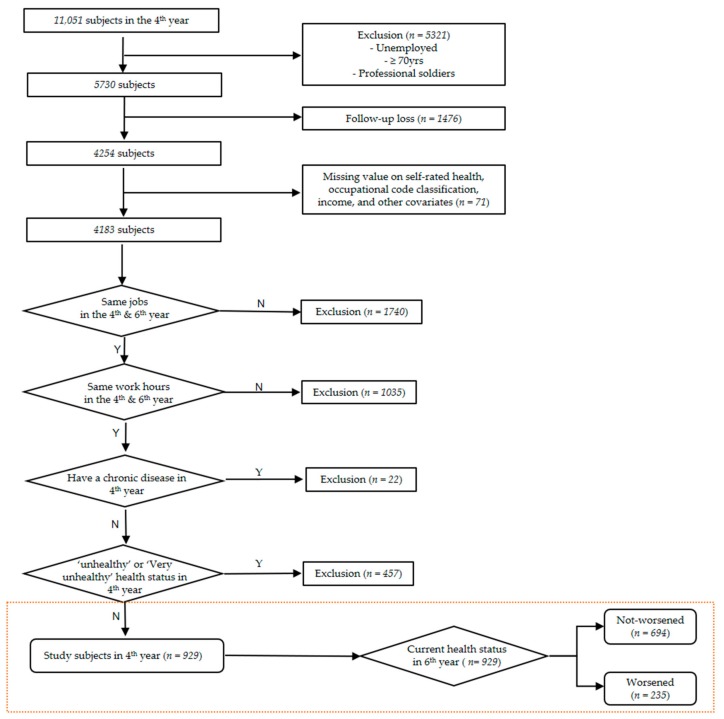
The flow chart of our research. The section marked with an orange box is intended to be confirmed through this study.

Among the 11,051 respondents in the 4th year, the 5321 subjects who were of unemployed status, over 70 years of age, or professional soldiers were excluded. Those who were not followed up in this survey, from the 4th year to the 6th year, were also excluded. The 4183 workers who had no missing values on self-rated health, occupation income, and other covariates were selected. In addition, we restricted the analyses to workers whose working hours and jobs had remained the same over the course of three years. To reduce the impact of the healthy worker effect, in the 4th year survey, we excluded 22 workers who were diagnosed with chronic diseases (cancer, stroke, myocardial infarction, or liver cirrhosis), and 457 workers who responded “unhealthy” or “very unhealthy” on self-rated health. As a result, a total of 929 workers were analyzed in the present study. Figure 1 shows our research flow chart. This study was approved by the Institutional Review Board of Ewha Womans University Mokdong Hospital (IRB approval number: EUMC 2018-07-077).

### 2.2. Working Hours

We used the working hours per week, which were defined as the summation of the regular working hours per one week and overtime working hours. The regular working hours were obtained from the question, “How many hours do you work in a week, excluding the meal time?” and overtime working hours were based on the question, “On average, how many hours do you work overtime per week?”. In this study, the working hours were categorized into a total of five groups by applying two steps. Firstly, the working hours were classified into four groups, based on the Korean Labor Standard Act: <40 h, 40–52 h, 53–68 h, and >68 h. The Korean labor standard act sets 40 h per week as legal working hours. Overtime work is possible up to 12 h per week, with the consent of the employee. In addition, it is also possible to work 16 h on weekends, apart from overtime work, so employees can work up to 68 h. Furthermore, according to Article 59 of the Korean Labor Standards Act, it is possible to work overtime of 12 h per week for 26 industries, such as transportation, communication, broadcasting, and health. It also enables some workers to work over 68 h a week. Secondly, the moving average was calculated as the average of the self-rated health scores for a total of nine working hours, including four working hours less and four working hours more than the base number of working hours. A dotted line graph was drawn of the moving average, and based on the graph, the 40–52 working hours group was additionally divided into two groups (40–46 h and 47–52 h). 

### 2.3. Self-Rated Health (SRH)

Participants in KLIPS were asked to self-rate their health on a 5-point scale. This scale was categorized as follows: “very healthy”, “healthy”, “moderate”, “unhealthy”, and “very unhealthy”. As mentioned before, we excluded those who answered “unhealthy” or “very unhealthy” in the 4th year survey. “Worsened” was defined as a decrease in health level from the 4th year of more than one step. Otherwise, it was classified as “not-worsened”

### 2.4. Socioeconomic Position

Occupations were categorized into 10 groups, based on the Korean Standard Occupation Classification. The household income was calculated as equivalized income using the equation (i.e., total household income ÷ √household size), and was divided into four groups by quartile. We categorized workers as permanent worker, temporary worker, and self-employed. The permanent workers were defined as workers who have wages and employment contracts with more than one year or have wages and employment contracts without fixed contract terms, which are possible to keep work. The waged-workers, who have employment contracts with less than one year, have daily salaries, or work as on-call workers, were categorized as temporary workers. Furthermore, the non-waged workers, who responded that they run their own business, were defined as self-employed. 

### 2.5. Statistical Analysis

We assessed distribution of working hours of study subjects depending on demographic characteristics, socioeconomic position, and occupational characteristics. We also confirmed the proportion of “not-worsened” and “worsened” health status, by the characteristics of study subjects.

Binominal logistic regression analysis was used to evaluate the associations between working hours and self-rated health, in terms of the prevalence ratio (PR). We constructed three models. In the crude model, only age was adjusted for assessing the PR. In model 1, further adjustment was performed for education level, employment status, job type, income, and marital status. In model 2, additional adjustment was made for self-rated health level in the 4th year. The estimated PR and 95% confidence intervals (CIs) were presented in all models. All statistical analyses were conducted using the SAS version 9.4 (SAS Institute Inc., Cary, NC, USA), and statistical significance was assessed by two-tailed *p* values of < 0.05.

## 3. Results

Table 1 summarizes the working hours per week, according to the general characteristics of the study subjects through the proportion. The mean working hours per week in women (49.5 h) was about four hours shorter than that in men (53.7 h). Most of the people in the group who were university-educated or more, worked between 40 to 46 h, in both men and women. In the self-employed and the sales workers, the proportion of the group working over 53 h was relatively high in both genders. In the marital status category, the widowed, divorced, and separated status showed that the percentage belonging to over 68 h was 29% in men and 36% in women. 

Table 2 shows the self-rated health status in the study subjects. There were 27.2% of men and 22.3% of women, who responded that their health status had worsened. As age increased, the proportion of worsened health status increased, and the higher level of education showed a higher proportion of the not-worsened health status only in women. The men who worked as temporary workers had the highest percentage of worsened health status (32.0%). Temporary female workers also showed the highest percentage of worsened health (26.9%). The proportion of worsened health was high in the group which was married, but with the spouse absent (widowed, divorced, and separated). Depending on the monthly household income level, the highest proportion of worsened health was in men with the highest income group. On the other hand, in women, the lowest income group had the highest percentage of worsened health status. Furthermore, the group of 40–46 working hours per week showed that the proportion of worsened health status was the lowest in women, whilst the men with less than 40 working hours had the lowest proportion of worsened health. The change in SRH of the study subjects are shown in the Supplementary Table (S1-1, S1-2)

The associations between working hours and worsened self-rated health are shown in Table 3. When the stratified analyses by gender were performed, we found that working hours per week of 47–52 h, 53–68 h, and >68 h in men were associated with 1.2, 1.3, and 1.1 times increases in the odds ratio of worsened self-rated health, compared with the reference group, respectively. On the other hand, in women, the odds ratio was 1.0, 2.2, and 2.6 times increases. However, in workers with less than 40 h, there were differences in the results of men and women. In other words, the men who worked less than 40 h had 0.9 times odds ratio of worsened self-rated health, and the women who worked the same hours had 5.4 times odds ratio of worsened self-rated health, compared with the reference group.

## 4. Discussion

We confirmed the associations between the working hours per week and change of self-rated health. Except for the group of less than 40 h, as the working hours increased, the odds ratio of worsened self-rated health increased in both men and women. The effect size of working hours on the deterioration of self-rated health was larger in women than men. However, in the group with less than 40 h, the negative association between working hours and self-rated health were shown only in women.

In men, workers with less than 40 working hours were likely to have good health status, and the associations were statistically significant. Previous studies showed that short working hours were not good for health [20,24,25]. However, these studies encountered methodological problems, including healthy worker effects. To minimize the healthy worker effects, we excluded from the study subjects who were not healthy in the baseline study sample. After overcoming the methodological problems in this way, short working hours were good for health in especially men. In addition, health could be influenced by cultural differences related to job demands, job control, and rewards. When working with similar job demand, the risk of poor health could be greater for men than women [25]. In another study, the association between work characteristics, including job demand and job control, and poor mental health was stronger among men in Japan, as well as in Europe [24]. Excessive overtime or perceived burden of work was associated with higher risk of physical symptoms in men than women [26]. The health effects from work factors can be more pronounced in men. Therefore, after minimizing the healthy worker effects, it is possible that the group of men who work less than 40 h is the best in their health status.

Women were found to have better health in the 40–46 working hours group, than in the less than 40 h group. These results were different from men. Women with less than 40 working hours may have a more insecure employment status than men [27]. In our study, temporary workers who worked less than 40 h, were 14% and 23% for men and women, respectively. Job insecurity is associated with poor health and is generally higher in women than in men [28,29]. Furthermore, the labor market is highly gender-segmented in South Korea [30,31], as well as in other countries [30,32]. Whilst women work mainly in the caretaking, sales, and service sectors, men work mainly in the manufacturing sector [33]. There is also a difference in the occupational positions by gender. Men, not women, dominate in managing [33]. Women with disadvantages in relation to their aforementioned work characteristics may experience more stress than men, even though the women worked less than 40 h. Work stress can deteriorate health status. There have been a lot of studies that showed stress is associated with self-rated health [34,35,36].

Our study also confirmed that women with long working hours (>52 h) were at marked risk of poor self-rated health. There may be several reasons for this result. The aforementioned women who work mainly in the sales and service sectors, were often referred to as “pink collar workers”. Pink collar workers and unskilled workers both have poorer physical and mental health, than those who are not in this category [37,38,39]. In addition, service sector workers also have long working hours. In South Korea, according to the national data on working hours by occupational group, the proportion of overwork (>52 h) in service workers was 12%, ranking third, preceded by machinery operators, and elementary occupations [40,41]. It was also confirmed in our study that 30% of women service workers worked more than 68 h. Secondly, there is still an earnings gap between men and women in the labor market in South Korea [31]. Moreover, women are also at a disadvantage, in terms of social insurance coverage and promotion opportunities [33]. Even among women, the labor market is differentiated. Low-wage women workers are mainly non-regular workers, and have a low educational background, older age, and low promotion opportunities. On the other hand, high-wage women workers tend to work as managers, professionals, full-time, and are highly educated [33]. The labor market generally limits rewards for women. Several studies have confirmed that unreasonable rewards for work lead to poor health status [42,43]. The study found that the number of women in management positions decreases with the increasing proportion of long working hours [44]. Thirdly, gender inequalities in household chores are still predominant in most countries. Among them, Korean women work five and seven times more for household member care and routine housework, respectively, than men do [45]. This gap is bigger than in European countries and the United States. Women experience a greater negative impact on their health than men, because of their struggle with work–family conflicts and the burden of household chores [20,46]. Work–family conflicts could increase in women workers who worked long hours [47]. There is also a study indicating that the perceived load from housework and paid work is a more important factor than the real time of total work in health status [26].

The associations between SRH and mortality have inconsistency by gender [48]. Some studies reported a stronger association in men than in women. Other studies presented little gender differences in the relationship between SRH and mortality [49]. Several studies have shown more powerful associations in women [50]. Furthermore, studies on the prediction of mortality in the middle level of SRH, such as “fair” or “moderate”, are also inconsistent among gender [51,52]. Determining their health may vary according to age, gender, and various social backgrounds [53]. A study using the National Health and Nutrition Examination Surveys of South Korea, reported that musculoskeletal disorders accounted for gender differences in the association between mortality and SRH [54]. Furthermore, another study indicated that women in South Korea had a higher risk than U.S. women regarding their SRH. These findings suggested that the traditional sex role or difference of social and cultural backgrounds in South Korea, led to a negative impact on women’s health [55]. The difference in SRH by gender may have been due to the unfavorable position of women in the Korea labor market. Therefore, the long-term follow-up study or the comparison of the same age group may be needed to understand gender differences in SRH [56].

This study has some limitations. The results were obtained using data from more than 10 years ago, so it may be slightly different from the current situation. Our study could not consider some risk factors that could affect health status beyond working hours, such as shift work, work load, work control, and household chores. In addition, SRH is a subjective measurement for health and has differences by gender. The understanding of gender differences in SRH is still controversial. Therefore, the associations using SRH, between working hours and health requires careful interpretation. Despite these limitations, this study is novel in some aspects. We attempted to overcome the healthy worker effect, which is considered as the limitation of previous studies. In addition, we confirmed that the effect of gender on the association between working hours and self-rated health might be different.

## 5. Conclusions

Working hours and self-rated health were related in our study. In men, the shortest working hours group had the best health status, compared with other groups. On the other hand, in women, the association between working hours and self-rated health was shown to be U-shaped, based on the 40–46 working hours group. The differences by gender could be attributed to the characteristics of the working environment to which they belong. It is necessary to clarify the association between working hours and self-rated health, and to acquire the consistency of results through further useful studies.

## Figures and Tables

**Table 1:** Working hours per week by socio-demographic characteristics in the study subjects.

N (%)													
		Men						Women					
		N	<40	40–46	47–52	53–68	>68	N	<40	40–46	47–52	53–68	>68
		571	25 (4.4)	154 (27.0)	117 (20.5)	184 (32.2)	91 (15.9)	358	29 (8.1)	142 (39.7)	88 (24.6)	56 (15.6)	43 (12.0)
Age	15~29	94	2 (2.1)	24 (25.5)	22 (23.4)	33 (35.1)	13 (13.8)	141	9 (6.4)	70 (49.7)	40 (28.4)	19 (13.5)	3 (2.1)
	30~39	197	5 (2.5)	39 (19.8)	52 (26.4)	78 (39.6)	23 (11.7)	78	12 (15.4)	34 (43.6)	22 (28.2)	5 (6.4)	5 (6.4)
	40~49	176	5 (2.8)	61 (34.7)	27 (15.3)	54 (30.7)	29 (16.5)	100	5 (5.0)	28 (28.0)	20 (20.0)	25 (25.0)	22 (22.0)
	50~59	88	10 (11.4)	28 (31.8)	14 (15.9)	18 (20.5)	18 (20.5)	24	1 (4.2)	7 (29.2)	3 (12.5)	6 (25.0)	7 (29.2)
	60~69	16	3 (18.8)	2 (12.5)	2 (12.5)	1 (6.3)	8 (50.0)	15	2 (13.3)	3 (20.0)	3 (20.0)	1 (6.7)	6 (40.0)
Education level	Less than elementary school	39	7 (18.0)	4 (10.3)	7 (18.0)	10 (25.6)	11 (28.2)	52	3 (5.8)	11 (21.2)	8 (15.4)	12 (23.1)	18 (34.6)
	Middle school	74	8 (10.8)	8 (10.8)	8 (10.8)	34 (46.0)	16 (21.6)	44	2 (4.6)	8 (18.2)	7 (15.9)	13 (29.6)	14 (31.8)
	High school	242	8 (3.3)	50 (20.7)	45 (18.6)	88 (36.4)	51 (21.1)	110	7 (6.4)	41 (37.3)	35 (31.8)	19 (17.3)	8 (7.3)
	College	51	0 (0.0)	12 (23.5)	14 (27.5)	19 (37.3)	6 (11.8)	58	2 (3.5)	27 (46.6)	20 (34.5)	9 (15.5)	0 (0.0)
	University or more	165	2 (1.2)	80 (48.5)	43 (26.1)	33 (20.0)	7 (4.2)	94	15 (16.0)	55 (58.5)	18 (19.2)	3 (3.2)	3 (3.2)
Employment status	Permanent worker	401	6 (1.5)	132 (32.9)	95 (23.7)	126 (31.4)	42 (10.5)	262	12 (4.6)	130 (49.6)	70 (26.7)	39 (14.9)	11 (4.2)
	Temporary worker	50	7 (14.0)	9 (18.0)	9 (18.0)	17 (34.0)	8 (16.0)	52	12 (23.1)	8 (15.4)	17 (32.7)	8 (15.4)	7 (13.5)
	Self-employed	120	12 (10.0)	13 (10.8)	13 (10.8)	41 (34.2)	41 (34.2)	44	5 (11.4)	4 (9.1)	1 (2.3)	9 (20.5)	25 (56.8)
Occupation	Managers	24	0 (0.0)	11 (45.8)	5 (20.8)	5 (20.8)	3 (12.5)	2	0 (0.0)	2 (100.0)	0 (0.0)	0 (0.0)	0 (0.0)
	Professionals	47	0 (0.0)	27 (57.5)	15 (31.9)	4 (8.5)	1 (2.1)	66	5 (7.6)	39 (59.1)	17 (25.8)	4 (6.1)	1 (1.5)
	Technicians	46	2 (4.4)	20 (43.5)	10 (21.7)	10 (21.7)	4 (8.7)	25	5 (20.0)	8 (32.0)	8 (32.0)	3 (12.0)	1 (4.0)
	Clerical workers	90	0 (0.0)	31 (34.4)	25 (27.8)	31 (34.4)	3 (3.3)	97	5 (5.2)	53 (54.6)	23 (23.7)	13 (13.4)	3 (3.1)
	Service workers	21	0 (0.0)	7 (33.3)	6 (28.6)	4 (19.1)	4 (19.1)	43	7 (16.3)	10 (23.3)	9 (20.9)	4 (9.3)	13 (30.2)
	Sales workers	48	5 (10.4)	4 (8.3)	9 (18.8)	19 (39.6)	11 (22.9)	39	1 (2.6)	10 (25.6)	5 (12.8)	8 (20.5)	15 (38.5)
	Skilled agriculture, forestry, and fishery workers	10	1 (10.0)	1 (10.0)	3 (30.0)	2 (20.0)	3 (30.0)	5	1 (20.0)	0 (0.0)	0 (0.0)	2 (40.0)	2 (40.0)
	Craft workers	143	11 (7.7)	27 (18.9)	20 (14.0)	50 (35.0)	35 (24.5)	25	1 (4.0)	4 (16.0)	10 (40.0)	9 (36.0)	1 (4.0)
	Machinery operators	104	3 (2.9)	20 (19.2)	17 (16.4)	44 (42.3)	20 (19.2)	37	1 (2.7)	7 (18.9)	13 (35.1)	12 (32.4)	4 (10.8)
	Elementary occupations	38	3 (7.9)	6 (15.8)	7 (18.4)	15 (39.5)	7 (18.4)	19	3 (15.8)	9 (47.4)	3 (15.8)	1 (5.3)	3 (15.8)
Household income(/month) unit: KRW	≤687,500	137	12 (8.8)	18 (13.1)	29 (21.2)	50 (36.5)	28 (20.4)	96	11 (11.5)	24 (25.0)	24 (25.0)	17 (17.7)	20 (20.8)
	687,500~1,000,000	167	7 (4.2)	36 (21.6)	32 (19.2)	60 (35.9)	32 (19.2)	78	3 (3.9)	22 (28.2)	23 (29.5)	16 (20.5)	14 (18.0)
	1,000,000~1,443,400	134	6 (4.5)	48 (35.8)	23 (17.2)	36 (26.9)	21 (15.7)	86	7 (8.1)	43 (50.0)	18 (20.9)	12 (14.0)	6 (7.0)
	>1,443,400	133	0 (0.0)	52 (39.1)	33 (24.8)	38 (28.6)	10 (7.5)	98	8 (8.2)	53 (54.1)	23 (23.5)	11 (11.2)	3 (3.1)
Marital status	Unmarried	99	5 (5.1)	24 (24.2)	25 (25.3)	37 (37.4)	8 (8.1)	131	10 (7.6)	66 (50.4)	35 (26.7)	16 (12.2)	4 (3.1)
	Married	455	18 (4.0)	127 (27.9)	89 (19.6)	143 (31.4)	78 (17.1)	199	16 (8.0)	69 (34.7)	51 (25.6)	34 (17.1)	29 (14.6)
	Widowed, divorced, and separated	17	2 (11.8)	3 (17.7)	3 (17.7)	4 (23.5)	5 (29.4)	28	3 (10.7)	7 (25.0)	2 (7.1)	6 (21.4)	10 (35.7)

**Table 2:** The change of self-rated health by socio-demographic characteristics in the study subjects.

		N (%)					
		Men			Women		
		Total	Not-Worsened	Worsened	Total	Not-Worsened	Worsened
		571	416 (72.8)	155 (27.2)	358	278 (77.7)	80 (22.3)
Age	15~29	94	69 (73.4)	25 (26.6)	141	121 (85.8)	20 (14.2)
	30~39	197	140 (71.1)	57 (28.9)	78	60 (76.9)	18 (23.1)
	40~49	176	127 (72.2)	49 (27.8)	100	76 (76.0)	24 (24.0)
	50~59	88	66 (75.0)	22 (25.0)	24	14 (58.3)	10 (41.7)
	60~69	16	14 (87.5)	2 (12.5)	15	7 (46.7)	8 (53.3)
Educational level	Less than elementary school	39	26 (66.7)	13 (33.3)	52	27 (51.9)	25 (48.1)
	Middle school	74	52 (70.3)	22 (29.7)	44	36 (81.8)	8 (18.2)
	High school	242	187 (77.3)	55 (22.7)	110	86 (78.2)	24 (21.8)
	College	51	35 (68.6)	16 (31.4)	58	48 (82.8)	10 (17.2)
	University or more	165	116 (70.3)	49 (29.7)	94	81 (86.2)	13 (13.8)
Employment status	Permanent worker	401	287 (71.6)	114 (28.4)	262	207 (79.0)	55 (21.0)
	Temporary worker	50	34 (68.0)	16 (32.0)	52	38 (73.1)	14 (26.9)
	Self-employed	120	95 (79.2)	25 (20.8)	44	33 (75.0)	11 (25.0)
Occupation	Managers	24	15 (62.5)	9 (37.5)	2	2 (100.0)	0 (0.0)
	Professionals	47	38 (80.9)	9 (19.2)	66	59 (89.4)	7 (10.6)
	Technicians	46	30 (65.2)	16 (34.8)	25	19 (76.0)	6 (24.0)
	Clerical workers	90	64 (71.1)	26 (28.9)	97	83 (85.6)	14 (14.4)
	Service workers	21	15 (71.4)	6 (28.6)	43	30 (69.8)	13 (30.2)
	Sales workers	48	37 (77.1)	11 (22.9)	39	28 (71.8)	11 (28.2)
	Skilled agriculture, forestry, and fishery workers	10	8 (80.0)	2 (20.0)	5	4 (80.0)	1 (20.0)
	Craft workers	143	105 (73.4)	38 (26.6)	25	14 (56.0)	11 (44.0)
	Machinery operators	104	71 (68.3)	33 (31.7)	37	28 (75.7)	9 (24.3)
	Elementary occupations	38	33 (86.8)	5 (13.2)	19	11 (57.9)	8 (42.1)
Household income(/month) unit: KRW	≤687,500	137	102 (74.5)	35 (25.6)	96	67 (69.8)	29 (30.2)
	687,500~1,000,000	167	122 (73.1)	45 (27.0)	78	57 (73.1)	21 (26.9)
	1,000,000~1,443,400	134	97 (72.4)	37 (27.6)	86	68 (79.1)	18 (20.9)
	>1,443,400	133	95 (71.4)	38 (28.6)	98	86 (87.8)	12 (12.2)
Workhours per week	<40	25	20 (80.0)	5 (20.0)	29	18 (62.1)	11 (37.9)
	40~46	154	113 (73.4)	41 (26.6)	142	120 (84.5)	22 (15.5)
	47~52	117	84 (71.8)	33 (28.2)	88	70 (79.6)	18 (20.5)
	53~68	184	131 (71.2)	53 (28.8)	56	41 (73.2)	15 (26.8)
	>68	91	68 (74.7)	23 (25.3)	43	29 (67.4)	14 (32.6)
Marital status	Unmarried	99	75 (75.8)	24 (24.2)	131	114 (87.0)	17 (13.0)
	Married	455	329 (72.3)	126 (27.7)	199	149 (74.9)	50 (25.1)
	Widowed, divorced, and separated	17	12 (70.6)	5 (29.4)	28	15 (53.6)	13 (46.4)

**Table 3:** Associations of working hours per week and worsened self-rated health.

Total	Men					Women				
	N	Case	Crude Model	Model 1	Model 2	N	Case	Crude Model	Model 1	Model 2
			PR (95% CI)	PR (95% CI)	PR (95% CI)			PR (95% CI)	PR (95% CI)	PR (95% CI)
	571	155				358	80			
Working hours per week										
<40	25	5	0.71 (0.70, 0.73)	0.73 (0.71, 0.75)	0.89 (0.86, 0.91)	29	11	3.68 (3.63, 3.74)	3.86 (3.79, 3.93)	5.35 (5.23, 5.47)
40~46	154	41	Ref			142	22	Ref		
47~52	117	33	1.14 (1.13, 1.15)	1.13 (1.12, 1.14)	1.19 (1.18, 1.21)	88	18	1.52 (1.50, 1.54)	1.19 (1.17, 1.20)	1.04 (1.02, 1.06)
53~68	184	53	1.22 (1.21, 1.23)	1.24 (1.23, 1.25)	1.28 (1.27, 1.30)	56	15	1.86 (1.83, 1.88)	1.62 (1.59, 1.65)	2.19 (2.15, 2.23)
>68	91	23	0.89 (0.88, 0.90)	0.98 (0.97, 1.00)	1.14 (1.13, 1.16)	43	14	2.13 (2.09, 2.16)	2.48 (2.43, 2.53)	2.64 (2.58, 2.70)

Crude model: adjusted for age, sex. Model 1: crude model + educational level, employment status, job type, household income, marital status. Model 2: Model 1 + previous self-rated health level.

## Supplementary Materials

The following are available online at http://www.mdpi.com/1660-4601/15/9/1984/s1, Table S1-1: The change of self-rated health in men, S1-2: The change of self-rated health in women.
