# Supplementary material for: The Change of Self-Rated Health According to Working Hours for Two Years by Gender
Source: Int J Environ Res Public Health. 2018 Sep 11;15(9):1984. doi: 10.3390/ijerph15091984 (PMC6164647; doi:10.3390/ijerph15091984)
Supplement: Supplementary file 1 [file ijerph-15-01984-s001.pdf]

Supplementary 1-1. The change of self-rated health in men

		SRH in 6th year					
		Very healthy	Healthy	Moderate	Poor	Very poor	Total
SRH in 4th year	Very healthy	4(8.3)	32(66.7)	11(1.9)	1(2.1)	0(0.0)	48(8.4)
	Healthy	30(7.3)	276(67.5)	92(22.5)	11(2.7)	0(0.0)	409(71.6)
	Moderate	7(6.1)	53(46.5)	46(40.4)	8(7.0)	0(0.0)	114(20.0)
	Total	41(7.2)	361(63.2)	149(26.1)	20(3.5)	0(0.0)	571(100.0)

Supplementary 1-2. The change of self-rated health in women

		SRH in 6th year					
		Very healthy	Healthy	Moderate	Poor	Very poor	Total
SRH in 4th year	Very healthy	2(11.8)	8(47.1)	6(35.3)	1(5.9)	0(0.0)	17(4.8)
	Healthy	17(6.8)	176(70.7)	50(20.1)	6(2.4)	0(0.0)	249(69.5)
	Moderate	2(2.2)	41(44.6)	40(43.5)	9(9.8)	0(0.0)	92(25.7)
	Total	21(5.9)	225(62.8)	96(26.8)	16(4.5)	0(0.0)	358(100.0)
